# GABA Levels in Left and Right Sensorimotor Cortex Correlate across Individuals

**DOI:** 10.3390/biomedicines6030080

**Published:** 2018-07-24

**Authors:** Nicolaas A. J. Puts, Stefanie Heba, Ashley D. Harris, Christopher John Evans, David J. McGonigle, Martin Tegenthoff, Tobias Schmidt-Wilcke, Richard A. E. Edden

**Affiliations:** 1 Russell H. Morgan Department of Radiology and Radiological Science, The Johns Hopkins University School of Medicine, 600 N Wolfe Street, Baltimore, MD 21287, USA; ashley.harris2@ucalgary.ca (A.D.H.); raee2@jhu.edu (R.A.E.E.); 2 F.M. Kirby Center for Functional Brain Imaging, Kennedy Krieger Institute, 707 N Broadway Street, Baltimore, MD 21205, USA; 3 Department of Neurology, BG University Hospital Bergmannsheil, Bürkle de la Camp-Platz 1, 44789 Bochum, Germany; Stefanie.Heba@rub.de (S.H.); martin.tegenthoff@ruhr-uni-bochum.de (M.T.); 4 Department of Radiology, University of Calgary, 1403-29th Street N.W., Calgary, AB T2N 2T9, Canada; 5 Child and Adolescent Imaging Research Program, Alberta Children’s Hospital Research Institute, University of Calgary, 2888 Shaganappi Trail, Calgary, AB T3B 6A8, Canada; 6 Hotchkiss Brain Institute, 3330 Hospital Drive NW, Calgary, AB T2N 4N1, Canada; 7 CUBRIC, School of Psychology, Cardiff University, Cardiff CF24 4HQ, Wales, UK; EvansJ31@cardiff.ac.uk (C.J.E.); mcgonigled@cardiff.ac.uk (D.J.M.); 8 School of Biosciences, Cardiff University, Cardiff CF10 3AT, Wales, UK; 9 Department of Clinical Neuroscience and Medical Psychology, Heinrich Heine University Düsseldorf, Universitätsstr. 1, 40225 Düsseldorf, Germany; tobias-schmidt-wilcke@t-online.de; 10 St. Mauritius Therapieklinik, 40670 Meerbusch, Germany

**Keywords:** GABA, MRS, sensorimotor, individual differences, bilateral inhibition

## Abstract

Differences in γ-aminobutyric acid (GABA) levels measured with Magnetic Resonance Spectroscopy have been shown to correlate with behavioral performance over a number of tasks and cortical regions. These correlations appear to be regionally and functionally specific. In this study, we test the hypothesis that GABA levels will be correlated within individuals for functionally related regions—the left and right sensorimotor cortex. In addition, we investigate whether this is driven by bulk tissue composition. GABA measurements using edited MRS data were acquired from the left and right sensorimotor cortex in 24 participants. T1-weighted MR images were also acquired and segmented to determine the tissue composition of the voxel. GABA level is shown to correlate significantly between the left and right regions (*r* = 0.64, *p* < 0.03). Tissue composition is highly correlated between sides, but does not explain significant variance in the bilateral correlation. In conclusion, individual differences in GABA level, which have previously been described as functionally and regionally specific, are correlated between homologous sensorimotor regions. This correlation is not driven by bulk differences in voxel tissue composition.

## 1. Introduction

Magnetic resonance spectroscopy (MRS) measurements can detect individual differences in the levels of γ-aminobutyric acid (GABA), which are correlated with behavioral paradigms thought to rely on the efficacy of the GABAergic system. These findings suggest that MRS can be used to investigate the role of GABA in brain function. Individual differences in GABA levels have been shown to correlate with: the size of the blood oxygen level dependent (BOLD) signal change in functional MRI experiments (an indirect measure of neuronal activity) [1,2,3,4,5]; functional responses to stimuli as recorded by magnetoencephalography [1,6]; psychophysical task performance [4,7,8,9,10]; quantitative measures of personality traits [11,12] and age [13]. These correlational relationships have been shown in various regions of the brain, including primary sensory and motor regions [4,8,9,14], prefrontal motor regions [7,10] and areas of the frontal lobe [11,13,15] and suggest that individual differences in GABA levels have functional and behavioral correlates.

Several of these studies have shown that functional metrics correlate with GABA levels in areas thought to underlie the behavioral performance being measured, and they do not correlate with GABA levels in other, unrelated cortical regions. For example, tactile discrimination performance correlates with GABA levels measured in a voxel centered on primary somatosensory cortex, but not with GABA levels in visual regions [9]. Similarly, eye movement control correlates with GABA levels measured in a voxel including frontal eye field (an important region for eye movement planning and execution), but not with GABA in an occipital voxel [10]. Thus individual differences in GABA appear to be both functionally relevant and region-specific. Two separate studies [11,16], studying a large number of voxels, have found no significant inter-regional correlations between five regions of interest. Thus, it appears that individual differences in GABA levels do not merely reflect global concentration, but also individual differences that underlie aspects of behavior.

To date no studies have examined individual differences in GABA levels in homologous regions in the healthy adult brain. While hemispheric asymmetries are known to exist for higher-order regions (e.g., areas involved in language such as Broca’s area), the somatosensory and motor cortices have similar structure and function between the hemispheres, and inhibition between bilateral motor cortex is important for motor control [17,18,19]. Anatomical and functional symmetries have been found [20,21,22], but neurochemical symmetries have not yet been explored. Understanding of the relationship between GABA levels in highly-connected, or homologous regions, is important for understanding health and disease. For instance, in conditions of plasticity, understanding how GABA in different regions interacts is of strong interest. In addition, hand-dominance has been shown to be associated with differences in interhemispheric inhibition [22,23,24], and therefore may associate with local inhibitory GABA levels.

Therefore, in this study we set out to examine whether GABA levels are correlated between spatially separate but functionally related brain regions: the left and right primary sensorimotor cortices (S1M1). GABA is more highly concentrated in grey matter (GM) than white matter (WM) [25], so we also explored whether individual differences in GABA level are driven by differences in voxel GM fraction. We also tested the extent to which they were driven by bulk tissue properties.

## 2. Results

**Figure 1:**
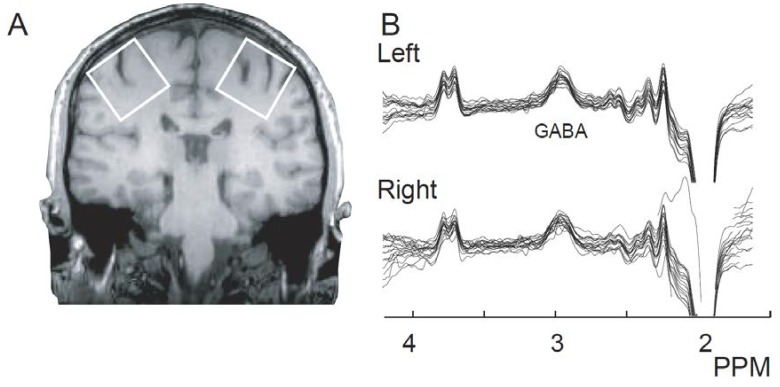
(**A**). Voxel locations. Single-participant example voxels over the left and right sensorimotor cortex. The center of the voxel was placed on the “hand knob”, an anatomical landmark indicating the hand area of the primary motor cortex, with the hand area of primary somatosensory cortex, directly posterior across the central sulcus, also included. The voxels are rotated to be aligned with the edge of the brain; (**B**). GABA-edited MR spectra from all participants for the left and right sensorimotor cortex. A high-quality GABA peak can be seen at 3 ppm for all participants.

Figure 1B shows all spectra from both the left and right sensorimotor cortex. We first assessed data quality to ensure that differences in quality between hemispheres did not drive the results. Data quality did not differ between left and right S1M1 (all fit errors <12%, mean fit errors 5.23 ± 1.99). Spectra are generally considered to be of sufficient quality when the fit errors are below 12% [26], as which is an arbitrary quantitative cut-off based on visual analysis of a large number of spectra. In addition, the fit errors presented here are consistent with those presented in previous work from a multi-site study of 24 sites [27] where average fit error was 5–6%, showing that the data presented here are of typical quality.

**Figure 2:**
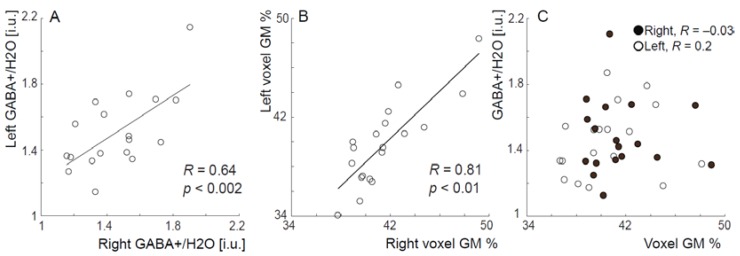
(**A**). Left and right sensorimotor GABA level (tissue-corrected) are correlated (*R* = 0.64, *p* < 0.002); (**B**). Voxel percentages of gray matter (GM%) are strongly correlated between left and right sensorimotor cortex across individuals; (**C**). GABA levels and %GM do not correlate. Therefore, %GM does not account for significant inter-individual variance in GABA level and the correlation (Figure 2A) is likely to be driven by biochemical differences, rather than by bulk differences in voxel tissue composition.

Results show that CSF-corrected GABA levels in left S1M1 were significantly correlated with GABA levels in right S1M1, as seen in Figure 2A (*R* = 0.64, *p* < 0.002; for tissue-uncorrected GABA values *R*= 0.57, *p* = 0.01). As shown in Figure 2B, the percentage of grey matter (GM%) in the two voxels was also highly correlated (*R* = 0.81, *p* < 0.0001), however GM% was not correlated with GABA levels for either the right or left side (Figure 2C), or across all data pooled (*R* < 0.25 and *p* > 0.21 for all three tests).

There was a trend towards correlations between left (dominant) GABA levels and the Edinburgh handedness quotient for the right (dominant) side (*r* = 0.44, *p* < 0.06, and between left GABA and the Edinburgh handedness quotient (*r* = 0.43, *p* < 0.07), but this was not statistically significant. There were no correlations with GABA measures on the non-dominant side (*R* = −0.19).

## 3. Discussion

We found a significant inter-region intra-individual correlation in GABA levels between homologous brain regions. Furthermore, although there is a difference in GABA level between gray and white matter [25] and voxel GM% is bilaterally correlated, voxel GM% did not explain a significant amount of the variance in GABA.

The lack of a correlation between GABA and GM% is not surprising, given the small variability we have in voxel composition. We therefore may not possess the statistical power to detect a correlation between GABA and tissue composition.

To date, few studies have examined whether homologous areas between the hemispheres (such as left vs. right S1M1) co-vary in anatomy and function across subjects. On the whole, studies have concentrated either on individual region-of-interest (ROI), or individual voxel examinations of differences. These studies [28] have focused on measuring absolute differences in grey matter volume or density. Voxel-based analysis of T1-weighted structural scans has shown that individual differences in voxel intensities, presumably reflecting grey-matter thickness, tend to co-vary between the left and right somatosensory regions [29]. Our own result of correlated GM% between sides reflects the same tendency.

Functional measures of interhemispheric effects have shown that there is also significant temporal covariance between the left and right sensorimotor cortex. Most notably, the emergence of resting-state functional MRI analysis was initiated by the observation that the temporal variations in signal intensity observed in the absence of an explicit task—at “rest”—show significant patterns of covariation between voxels in the left and right motor cortex [30]. The same has been shown to be true of left and right sensorimotor cortex [31,32,33]. Our measure of a further level of organization, neurotransmitter level, has demonstrated a significant covariance of GABA levels bilaterally in a region incorporating both somatosensory and motor cortices, across subjects. While it may seem obvious that GABA in homologous regions is closely related given the previous findings showing anatomical and functional correlations, we show here that such a correlation is exhibited on a neurochemical level, independent from gross structural homologues. As no significant inter-regional correlations were found in previous studies between MRS regions that are less closely related functionally, we believe that our results demonstrate that there are neurochemical “signatures” of brain anatomy, potentially complementary to functional or structural measures [34].

What mechanisms might underlie co-varying neurotransmitter levels between one hemisphere with a homologous region in the other hemisphere? As noted in reference [29], genetic and epigenetic, use-dependent mechanisms could explain the results that we see in the current study. It is possible that the observed correlation reflects balanced, mutual interhemispheric inhibition. Evidence of such interhemispheric interactions has been shown by electrophysiology [35,36]. In spite of the fact that sensory processing is dominantly contralaterally located, there is evidence of inter-hemispheric connectivity and bilateral processing in both the motor and somatosensory domain. Recent work (reviewed in reference [20]) has suggested that these may be bilateral representations of the body surface in S1, and optical imaging in non-human primates [21] has demonstrated that both ipsilateral and contralateral skin stimulation affect the response of contralateral SI.

The MEGA-PRESS technique applied here to measure GABA has a number of limitations. In order to acquire sufficient signal-to-noise, a voxel size of ~27 mL per 10-min acquisition (or an equivalent size-to-scan-time trade-off) is typically required [37]. Measurement voxels are also limited to a cuboidal geometry. Therefore the regions interrogated incorporate both somatosensory and motor cortices. Novel approaches, such as MEGA-PRIAM [38], allow for simultaneous acquisition of regions such as right and left S1M1, but this technique needs further validation. In addition, the edited GABA peak contains co-edited macromolecular signal and is often referred to as GABA+ for this reason. From the experiments presented, it is likely that inter-individual differences in MM contribute to the observed effects.

Brain chemistry gives rise to brain connectivity, and brain activity, which in turn give rise to behaviors. While functional connectivity is a well-studied area, there is little understanding how homologous, or tightly connected, regions are associated in terms of neurochemistry. Differences in connectivity may give rise to neurological problems (e.g., altered motor control in neurodevelopmental disorders) [39,40] which may be associated with differences in neurochemistry. Cortical rehabilitation plasticity, e.g., after stroke, often requires recruitment of contralateral homologous regions. A better understanding of the neurochemical relationship may provide with more data to study baseline neurochemistry, as well as future work targeting therapies, recovery and learning.

## 4. Materials and Methods

### 4.1. Participants

Our cohort consisted of 24 healthy right-handed participants aged 23.8 ± 3.5 years (8 female), scanned with local ethics committee approval and written informed consent. Handedness was assessed using the Edinburgh handedness inventory [41] and reported as handedness quotient ((R − L)/(R + L)) × 100) which reflects a bias towards using the right hand. All participants were right-handed (mean score: 20 ± 3.09, mean quotient 67.38 ± 25.16).

### 4.2. MEGA-PRESS

All GABA-edited MRS data were acquired using the MEGA-PRESS technique [42]. Conventional single-voxel MRS does not allow for the reliable quantification of GABA levels due to significant overlap by larger, higher-concentration metabolites such as creatine. In MEGA-PRESS, the GABA signal is selectively manipulated in half of the transients by applying a GABA-specific editing pulse at 1.9 ppm (edit-ON) which selectively refocuses the GABA signal at 3 ppm. In the other half of the experiment the editing pulse is applied elsewhere, such that it does not affect the spectrum. The difference between the ON and OFF transients only shows those signals affected by the editing pulse, removing unwanted signal (creatine) from the spectrum and revealing GABA signal at 3 ppm.

### 4.3. Acquisition

T1-weighted MPRAGE images were acquired in each subject (repetition time (TR) 8.5 ms, echo time (TE) 3.9 ms, flip angle 8 deg, voxel size (1 mm)^3^, FOV 256 × 256 × 220 mm)), prior to MRS. MEGA-PRESS GABA measurements were acquired in two sensorimotor volumes (3 × 3 × 3 cm^3^, Figure 1A) for each subject using a 3T Philips Achieva MRI scanner, without repeat per voxel. The voxels were centered on the ‘hand knob’ region of the motor cortex and rotated to align with the cortical surface [9,43]. Sequence parameters were: TE/TR 68/2000 ms; 14 ms sinc-Gaussian editing pulse applied alternately at 1.9 and 7.46 ppm (ON and OFF experiments); 320 transients; 2 k datapoints; 2 kHz spectral width, VAPOR water suppression. In order to perform the bilateral measurements symmetrically, the water-fat shift direction associated with the left-right and head-foot slice selection were reversed for the left hemisphere (relative to the right).

### 4.4. Data Processing

All MRS data were analyzed using Gannet software [26], programmed in MATLAB (The Mathworks, Natick, MA, USA). Frequency and phase correction were performed using Spectral Registration [44]. GABA levels in ‘institutional units’ were quantified from the ratio of the integral of the edited GABA signal (determined by fitting to a Gaussian model) to the integral of the unsuppressed water signal from the same volume (determined by fitting to a Lorentzian-Gaussian model) and a constant multiplier used to account for differences in T_1_ and T_2_ relaxation times of water and GABA and the editing efficiency [25,45]. Model fit error was assessed by normalizing the SD of the fit residuals to the amplitude of the respective modeled signal (GABA and water). Overall fit error was then defined as the root sum of squares of the GABA and water fit errors.

Co-registration of the MRS voxel position to the T1-weighted image and segmentation of the image was performed using FAST [46], which allowed the tissue composition to be expressed as percentage gray matter (GM%), white matter (WM%) and cerebrospinal fluid (CSF%). GABA levels were CSF-corrected to account for the fraction of the voxel in each subject that is CSF (and therefore contains no GABA). Data from 6 out of 24 recruited subjects were excluded due to one of the following reasons: medication with central modes of action (1), Beck Depression Index scores higher than 18 (1), hand injuries (1), poor-quality water reference data, i.e., water scans with more than two repeats rejected due to poor fitting (1) and movement artifacts larger than 3 mm translation or 3-degree rotation during MRS sessions (2), thus leaving data sets from eighteen subjects for final analyses.

### 4.5. Statistical Analysis

All statistical analyses were carried out in MATLAB. To test the hypothesis that there is a positive bilateral correlation of GABA levels, the Pearson correlation coefficient R between left and right S1M1 GABA (and the associated *p* value testing the null hypothesis) was calculated using Pearson correlations. Data quality per region was assessed by calculating the fitting error for each GABA spectrum, and left- and right differences were tested using a univariate analysis with fit error as dependent variable and hemisphere as fixed factor.

Correlational analysis was performed to test the hypotheses that left and right voxel GM fractions would be correlated between individuals, and that voxel GM% would be correlated with GABA level. Finally, it was assessed whether GABA levels for the dominant side (left S1M1) and non-dominant side (right S1M1) correlated with the Edinburgh handedness quotient.

## 5. Conclusions

In conclusion, we have shown that GABA levels are significantly correlated between the left and right sensorimotor regions, a correlation that is not driven by inter-individual differences in bulk gray matter content. These results show a significant inter-region correlation in GABA levels across symmetrically positioned, homologous regions.
